# The Importance of Cardiac Computed Tomography in the Diagnosis of Caseous Calcification of the Mitral Annulus—Case Reports

**DOI:** 10.3390/diagnostics12030667

**Published:** 2022-03-09

**Authors:** Paweł Gać, Przemysław Cheładze, Rafał Poręba

**Affiliations:** 1 Centre for Diagnostic Imaging, 4th Military Hospital, Weigla 5, PL 50-981 Wroclaw, Poland; przemdi@gmail.com; 2 Department of Population Health, Division of Environmental Health and Occupational Medicine, Wroclaw Medical University, Mikulicza-Radeckiego 7, PL 50-368 Wroclaw, Poland; 3 Department of Internal and Occupational Diseases, Hypertension and Clinical Oncology, Wroclaw Medical University, Borowska 213, PL 50-556 Wroclaw, Poland; rafal.poreba@umw.edu.pl

**Keywords:** cardiac computed tomography, caseous calcification of the mitral annulus, mitral annular calcification

## Abstract

Mitral annular calcification (MAC) is a common pathology of the mitral valve. In rare cases, calcifications occur in the mitral annulus degenerate serous; the caseous calcification of the mitral annulus (CCMA) then develops. Detection of CCMA is often random and requires differentiation from heart tumors or an abscess. The paper presents two cases of patients with ambiguous focal lesions of the mitral valve in echocardiography. In the first case, the cardiac computed tomography (CCT) showed a spherical, slightly irregular structure measuring approximately 33 × 22 mm, which was in contact with the posterior mitral valve leaflet from the lumen of the left ventricle. The lesion was heterogeneously intense, with an average density of about 500 HU and up to 975 HU on the periphery; it was not enhanced after the administration of a contrast agent. In the second case, the CCT revealed a heterogeneous, highly calcified structure in the peripheral zone and intermediate density in the central zone in the topography of the posterior mitral valve leaf, with dimensions up to about 41 × 31 mm in the plane of the valve leaflet, passing into the lumen of the left ventricle along its inferolateral wall to a depth of about 3.5 cm. In both cases, CCT enabled the diagnosis of CCMA. In conclusion, cardiac computed tomography may be decisive in the case of suspected caseous calcification of the mitral annulus where there is ambiguous echocardiography.

## 1. Introduction

Mitral annular calcification (MAC) is a common degenerative condition of the mitral valve. MAC affects approximately 8.5% of the general population [1,2]. Calcification is caused by a build-up of calcium in the annulus of the valve, leading to mitral regurgitation, or more rarely to narrowing or to a heart block. When calcified areas undergo caseous degeneration, a rare MAC variant appears, called caseous calcification of the mitral annulus (CCMA). The disease most often occurs in elderly patients, affecting mainly women [2], especially those at risk of cardiovascular diseases (diabetes, hypertension, obesity, renal failure, ischemic heart disease, and impaired calcium metabolism) [3]. It rarely occurs in younger patients—e.g., in people with Barlow’s disease [4] and Marfan’s syndrome [5]. It is currently estimated that it affects 0.068% of the general population and 0.64% of all MAC cases [6].

Caseous calcification of the mitral annulus usually presents as a round or crescent-shaped soft mass with central clearances, which is often seen in transthoracic echocardiography (TTE) and transesophageal echocardiography (TEE) and is very well visible in cardiac computed tomography (CCT) and cardiac magnetic resonance (CMR). Most of the described cases concern the posterior part of the mitral ring, while lesions in the anterior or the entire ring are less frequently reported [1,3].

CCMA is usually asymptomatic or mild, and its detection is often incidental and misdiagnosed as a heart tumor or abscess. Sometimes, however, it causes symptoms such as conduction abnormalities or causes systemic embolization with caseous material.

This report presents two cases of caseous calcification of the mitral annulus, aiming to emphasize the diagnostic importance of cardiac computed tomography in the assessment of heart valve morphology and the differentiation of tumor-like lesions of the heart.

## 2. Case Reports

### 2.1. Patient No. 1

This was a 76-year-old patient with diagnosed arterial hypertension, treated for glaucoma after a total thyroid resection due to malignant glaucoma. They were admitted to the Department of Cardiology, in good general condition, due to suspected myxoma or other neoplastic lesions in the mitral valve after TTE in an outpatient setting. Reported complaints included weakness and decreased capacity for physical activity.

After the patient was admitted to the Department of Cardiology, the TTE was performed again, showing a spherical, intensely saturated structure with a diameter of about 2 cm in the posterior pole of the mitral ring, with slight lightening in the central part. The formation seemed to be stably attached to the annulus and posterior leaflet of the mitral valve on the left atrium side. It was suggested that MAC was a possibility, and the diagnosis was extended to include TEE. In addition to the lesions described above, the study revealed mild aortic regurgitation, moderate tricuspid regurgitation, and regional contractility abnormalities in the lesion found.

Through TEE, the presence of myxoma was excluded, and there was a trace of mobility of the posterior mitral valve leaflet supported by calcification from the left ventricle. The nature of the lesion was not resolved, which was associated with the decision to refer the patient for a CCT.

**Figure 1:**
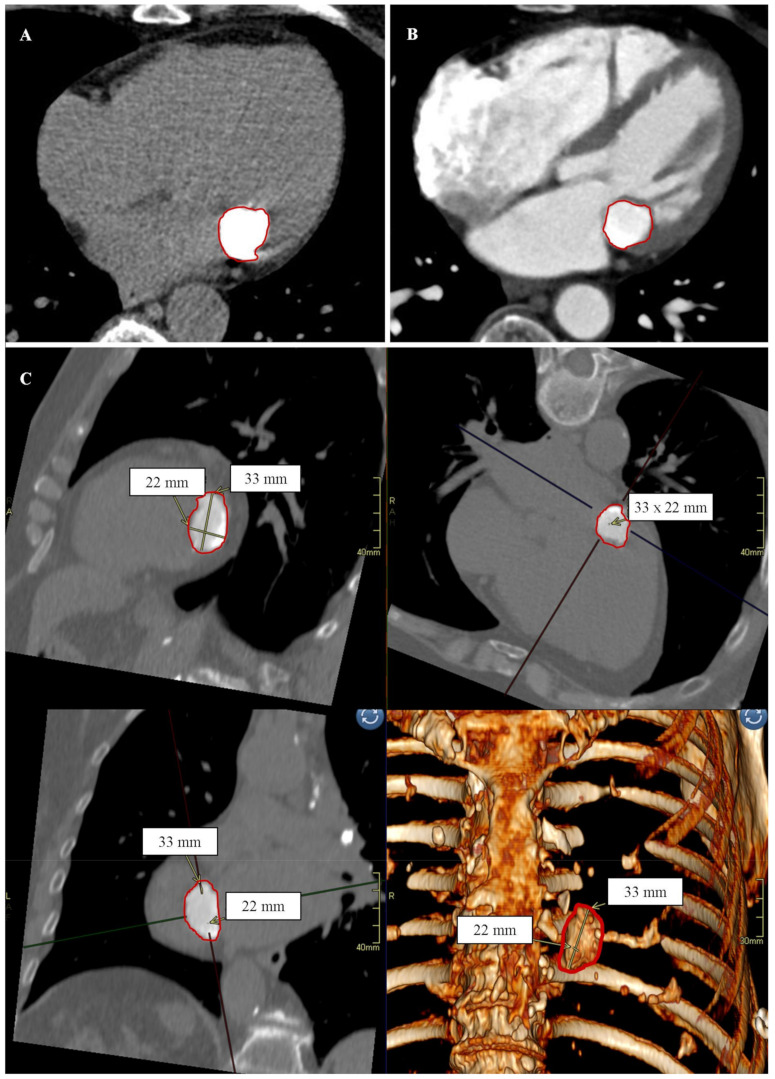
Patient No. 1. Caseous calcification of the mitral annulus (CCMA) in cardiac computed tomography. (**A**) Native phase. Axial reconstruction. The borders of the CCMA are marked in red. (**B**) Post-contrast phase. Axial reconstruction. The borders of the CCMA are marked in red. (**C**) Post-contrast phase. Secondary reconstructions. From the top and left side sequentially: MPR reconstruction, view in the short axis; MPR reconstruction, view in the long axis, 4-chamber; MPR reconstruction, view in the long axis, 2-chamber; VRT reconstruction. CCMA dimensions are marked with yellow lines and arrows; CCMA borders are marked in red.

The CCT showed a spherical, slightly irregular structure measuring approximately 33 × 22 mm, which was in contact with the posterior mitral valve leaflet from the lumen of the left ventricle. The lesion was heterogeneously intense, with an average density of about 500 HU and up to 975 HU on the periphery; it was not enhanced after the administration of a contrast agent. The images in the CCT allowed for the diagnosis of CCMA, Figure 1.

The patient remains under observation. There was no progression in the echocardiographic assessment in the 1-year perspective.

### 2.2. Patient No. 2

This patient was a 78-year-old woman presenting with atrial fibrillation and symptoms of CCS (Canadian Cardiovascular Society) class III angina during the routine check on the condition of a 2-lead pacemaker showing a low battery level. To replace the pacemaker, the patient was admitted for a one-day procedure to the Department of Cardiology, where a TTE was routinely performed. Through TTE, in addition to the features of moderate mitral and tricuspid valve regurgitation, significant thickening of the mitral ring and a mass of 2.9 × 1.8 cm in the lower pole projection, which included the basal inferior and anterolateral walls of the heart and convexed to the side of the heart, was visible.

After replacing the pacemaker battery, the symptoms of angina pectoris were partially resolved to class II on the CCS scale.

To further diagnose the suspicious lesion, a TEE and a CCT were performed as part of the one-day admission procedure.

In the TEE, in addition to the features of moderate mitral and tricuspid valve regurgitation, massive calcifications in the mitral ring forming a conglomerate approximately 2.0 × 1.1 cm from the pole side of the lower ring were discernible—an image suggestive of MAC. 

**Figure 2:**
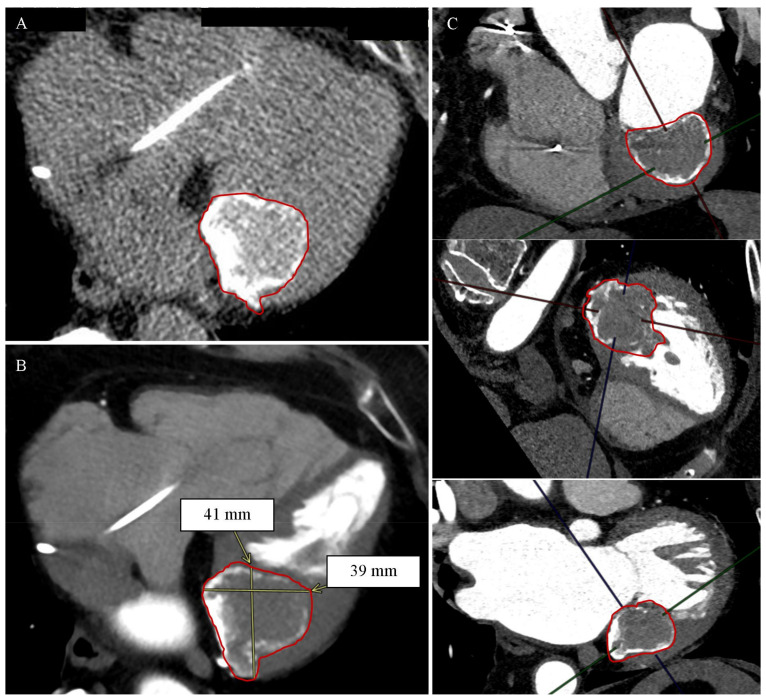
Patient No. 2. Caseous calcification of the mitral annulus (CCMA) in cardiac computed tomography. (**A**) Native phase. Axial reconstruction. The borders of the CCMA are marked in red. (**B**) Post-contrast phase. Axial reconstruction. CCMA dimensions are marked with yellow lines and arrows; the borders of the CCMA are marked in red. (**C**) Post-contrast phase. Secondary reconstructions. From the top sequentially: MPR reconstruction, view in the short axis; MPR reconstruction, view in the long axis, 4-chamber; MPR reconstruction, view in the long axis, 2-chamber. CCMA borders are marked in red.

The CCT revealed a heterogeneous, highly calcified structure in the peripheral zone and intermediate density in the central zone in the topography of the posterior mitral valve leaf, with dimensions up to about 41 × 31 mm in the plane of the valve leaflet, passing into the lumen of the left ventricle along its inferolateral wall to the depth of about 3.5 cm—the CCT image indicates CCMA, Figure 2.

This patient is also under observation. In the control TTE after 8 months, there was no significant change in the size of the lesion.

## 3. Discussion

Based on the studies conducted so far, the incidence of CCMA is considered low, ranging from 0.06% to 0.07% in the entire population, and up to 0.6% of patients with MAC. On the other hand, the frequency of occurrence in autopsies is higher and amounts to approximately 8% of the studied population. This condition is therefore probably being underdiagnosed due to its asymptomatic course in most patients [7].

Previous reports have shown that TTE was considered the most reliable method of diagnosing this disease. Caseous degeneration of the mitral flap manifests itself as a large, round mass with a dense echo and smooth borders in the pericardial region. Additionally, it should not have an acoustic shading effect. It was reported to be usually posterior to the left ventricle and bulging into the left atrium or the posterior wall of the left ventricle. With limited acoustic visibility or low-quality echocardiographic images, patients were referred for TEE, which has so far provided good visualization of the posterior structures. In both examinations, CCMA does not flow in the central zone in color Doppler and is surrounded by a hyperechoic structure [8]. When in doubt in the TTE, it is the CCT that leads to the final diagnosis [6]. In computed tomography, CCMA appears as a well-defined, oval or crescent-shaped, hypo-, iso- or hyperdense mass with peripheral calcifications of high Hounsfield units value, without contrast enhancement, usually located along the posterior mitral ring [6,8]. In case of doubt, and when CCT is still inconsistent with a diagnosis, cardiac magnetic resonance is the technique of choice. The CCMA results in CMR include a well-delimited mass with a hyperintense center and a hypointense edge separated from the myocardium and posterior mitral valve in T1-weighted imaging with fast spin-echo. In CMR T2-weighted sequences, CCMA appears as a mass devoid of a central signal, but with a ring of high intensity compared to the surrounding myocardium [8]. In the case of the above-described patients, both TTE and TEE did not give a definite diagnosis. The patients were referred for a CCT examination. The CCT image was highly suggestive for CCMA.

The presented cases indicate CCT as a diagnostic method enabling early and precise diagnosis of CCMA. They provide data for the ongoing discussion on the diagnostic accuracy of various imaging methods. Additionally, other advantages of using CCT as a CCMA imaging method should be highlighted. They include much better spatial resolution independent of the acoustic window and better tissue resolution compared to TTE, the non-invasive feature of the diagnostic procedure compared to TEE, significantly shorter examination time, better availability, and lower economic cost compared to CMR. The presented images were obtained using one of the most advanced computed tomography scanners currently available—384 (2 × 192) slices Siemens Somatom Force (Erlangen, Germany). Such a computed tomography scanner enables the acquisition of images in layers of 0.6 mm thickness, with a time resolution of <0.3 s, with a low dose of ionizing radiation <1 mSv and a low volume of contrast agent <60 mL [9]. CCT in the case of CCMA is a highly accurate method of differentiating from proliferative and inflammatory changes in the heart valves. The lack of post-contrast enhancement of the soft tissue lesion makes it possible to exclude neoplastic etiology, while the lack of a connective tissue capsule with post-contrast enhancement and the lack of gas within the lesion make it possible to exclude a paravalvular abscess [10,11].

In the natural history of all types of MAC, a slow progression in the severity of the lesions is observed [8]. In the presented cases, there was no progression of CCMA. This may be due to the short follow-up time.

Calcification of the mitral annulus has been associated with the risk of stroke (especially CCMA), infective endocarditis, and atrial fibrillation. Increasing the thickness of the lesions by 1 mm resulted in a 10% increase in the risk of stroke [12,13]. In almost 20% of patients operated on, for this reason, bacterial endocarditis was demonstrated [14]. The predictive value of this pathology in relation to the incidence and severity of coronary artery disease has been demonstrated [15]. The relationship of calcification of the mitral annulus with a three-times increased mortality in patients with chronic kidney disease has been shown [16].

There is no standard CCMA treatment protocol. Surgery is indicated in the event of severe mitral valve dysfunction (related primarily to other diseases). Surgical management has been implemented with great success in cases of CCMA with severe symptomatic mitral regurgitation, stenosis, or systemic embolization of the caseous material [1]. CCMA is more frequently surgically resected than typical MAC due to the higher prevalence of systolic embolization [17]. For most patients, 5-year survival after surgery is estimated at 76% [18]. However, most patients do not require surgery and are treated conservatively. Cases of spontaneous resolution or return to the typical MAC pattern have been described [8,19,20]. There have also been reports of CCMA recurrence even after surgical resection [1].

The limitation of the present case presentation is the lack of images from other diagnostic methods. Unfortunately, no images were recorded during the TTE and TEE examinations. Due to the final diagnosis in the CCT method, no further diagnostic procedures (CMR, PET-CT) were performed.

## 4. Conclusions

Cardiac computed tomography may be decisive in the case of suspected caseous calcification of the mitral annulus where there is ambiguous echocardiography.

## Data Availability

Not applicable.
